# Rockefeller, the Philanthropist

**Published:** 1908-01

**Authors:** 


### ROCKEFELLER, THE PHILANTHROPIST.

Everybody abuses Rockefeller. It's the fashion,—because he has made an immense fortune. I file a brief for the defense; not of Standard Oil, but of this alleged *hard* old fellow. He will live in history as "one who loved his fellow man"—like Ben Adhem; but, unlike Ben, he is one of the world's benefactors. It was a benefaction to distribute coal oil all over the world, to put it in reach of the poorest. Who minds a few cents more or less for a gallon of lamp oil? I remember that, when a boy, I used to get my lessons by a single "star" candle; my father got his by home-made tallow "dips," and he used to say that *his* father got his by "light-'ood"; that is, pine knots split up. Our first lamps burned lard oil, the next were "camphene" and "phosgene," as explosive as dynamite. The introduction of coal oil as a luminant was a distinct advance, and a blessing to millions. Well, Rockefeller made it possible for poor people. Read the "Song of the Shirt." Think what a luxury a coal oil lamp would have been to her who, with a single thread, "sewed at once a shirt and a shroud."

But Rockefeller gave $50,000,000 to education at one pop. Peabody isn't in it a little bit, nor Carnegie, either.

These things, however, I did not intend to allude to. What was in my mind to say was that Rockefeller gave New York City five millions to found an "Institute for Medical Research." This institute is now in operation, and one of the very first—if not the first—discoveries by "research" made possible by this misunderstood and abused old gentleman, is one of inestimable value and far-reaching importance. It is no less than that a small quantity of Epsom salts, hypodermically administered, is an anesthetic, local and general, void of the dangers that attend chloroform, ether and cocaine. And this discovery was made by experiment on a dog; perhaps, a "black beauty" or a "fido," whose sorrows are sung by the sentimental and hysterical and compared to which those of Werter, or of Rachel, or of Nioba were trifles. Now, mind you, I love a dog, and I may say as my friend Frank Johnson used to say, the more I see of some men the better I like dogs. *"Not* Cæsar less but Rome more." I love humanity more; and while the necessity of animal experimentation in the laboratory for the advancement of medical science and the prolongation of life is to be deplored, still it is necessary. In another part of this issue I reproduce an announcement of this great discovery, from a Metropolitan daily. Read it. If further developments confirm these statements, Metzler's name will go down to posterity with that of Simpson, Guthrie, Crawford Long, and Koller, the discoverer of cocaine anesthesia.

And, yet, notwithstanding this discovery; notwithstanding all the life-saving and pain-preventing agencies in the hands of the medical profession are the result of discoveries in laboratory research on the lower animals, the fanatics are at work at Albany to prevent the world from getting the benefit of Rockefeller's last donation, the $5,000,000 for laboratory research in animal experimentation.

